# ZBP-89 reduces histone deacetylase 3 by degrading IkappaB in the presence of Pin1

**DOI:** 10.1186/s12967-015-0382-7

**Published:** 2015-01-27

**Authors:** Cai Guo Ye, Liping Liu, George G Chen, Xiao Lin Tang, Zhiwei He, Ming-Liang He, Paul BS Lai

**Affiliations:** Department of Surgery, The Chinese University of Hong Kong, Prince of Wales Hospital, Shatin, Hong Kong, NT P. R. China; Department of Medicine and Therapeutics, The Chinese University of Hong Kong, Shatin, Hong Kong, NT P. R. China; Stanley Ho Centre for Emerging Infectious Diseases, The Chinese University of Hong Kong, Shatin, Hong Kong, NT P. R. China; Sino-America Cancer Research Institute, The Guangdong Medical College, Dongguan, Guangdong province P R China; Department of Hepatobiliary and Pancreas Surgery, the Second Clinical Medical College of Jinan University (Shenzhen People’s Hospital), Shenzhen, Guangdong Province China

**Keywords:** ZBP-89, HDAC3, Pin1, IκB, Hepatocellular carcinoma

## Abstract

**Background:**

Histone deacetylase 3 (HDAC3) is overexpressed in cancers and its inhibition enhances anti-tumor chemotherapy. ZBP-89, a transcription factor, can induce pro-apoptotic Bak and reduce HDAC3 but the mechanism is unknown. Pin1, a molecular switch that determines the fate of phosphoproteins, is known to interact with HDAC3. The aim of this study was to investigate the mechanism how ZBP-89 downregulated HDAC3.

**Methods:**

In this study, liver cells, Pin1-knockout Pin1^−/−^ and Pin1 wild-typed Pin^+/+^ cells were used to explore how ZBP-89 reduced HDAC3. The overexpression of ZBP-89 was achieved by infecting cells with Ad-ZBP-89, an adenoviral construct containing ZBP-89 gene. The role of NF-κB was determined using CAY10576, MG132 and SN50, the former two being inhibitors of IκB degradation and SN50 being an inhibitor of p65/p50 translocation. A xenograft tumor model was used to confirm the *in vitro* data.

**Results:**

ZBP-89 reduced HDAC3, and it could form a complex with IκB and induce IκB phosphorylation to inhibit IκB. Furthermore, ZBP-89-mediated HDAC3 reduction was suppressed by IκB degradation inhibitors CAY10576 and MG132 but not by p65/p50 translocation inhibitor SN50, indicating that IκB decrease rather than the elevated activity of NF-κB contributed to HDAC3 reduction. ZBP-89-mediated HDAC3 or IκB reduction was significantly less obvious in Pin1^−/−^ cells compared with Pin1^+/+^ cells. In Ad-ZBP-89-infected Pin1^+/+^ cancer cells, Pin1 siRNA increased HDAC3 but decreased Bak, compared with cells without ZBP-89 infection. These findings indicate that Pin1 participates in ZBP-89-mediated HDAC3 downregulation and Bak upregulation. The cell culture result was confirmed by *in vivo* mouse tumor model experiments.

**Conclusions:**

ZBP-89 attenuates HDAC3 by increasing IκB degradation. Such attenuation is independent of NF-κB activity but partially depends on Pin1. The novel pathway identified may help generate new anti-cancer strategy by targeting HDAC3 and its related molecules.

**Electronic supplementary material:**

The online version of this article (doi:10.1186/s12967-015-0382-7) contains supplementary material, which is available to authorized users.

## Background

ZBP-89, a Kruppel-like zinc finger binding transcription factor, binds GC-rich promoter elements to activate or suppress gene expression. ZBP-89 regulated expression can result in altered cell growth and death. Previously, we found that ZBP-89 could bind to histone deacetylase 3 (HDAC3) protein to inhibit its deacetylation activity, leading to the increase of pro-apoptotic Bak protein expression in hepatocellular carcinoma (HCC) [1,2]. HDAC3, a histone deacetylase enzyme, removes acetyl-residues from histones and forms transcriptional co-repressors with other proteins. HDAC3 can also act as a non-histone deacetylase [3,4], playing an important role in physiologic and pathological conditions, such as apoptosis, tumorigenesis and metastasis. The high expression of HDAC3 has been documented in HCC and associated with the reduced recurrence-free survival [5-7]. The inhibition of HDAC3 can lead to a strong anti-tumoral effect, which enhances the efficacy of chemotherapeutic agents in HCC [5,7]. Furthermore, the increase in HDAC3 may contribute to the hapatocarcinogenesis [6].

Peptidyl-prolyl cis/trans isomerase 1 (Pin1), a highly conserved enzyme, specifically phosphorylates Ser/Thr-Pro to control the fates of phospho-proteins [8]. For example, Pin1 can regulate the phosphorylation or dephosphorylation of proteins to prevent or enhance protein degradation and thus control diverse cellular processes including tumorigenesis. In HCC, Pin1 can form a complex with hepatitis B virus x protein (HBx), a well-known hepatocarcinogenetic factor, and overexpress in cancer tissues, indicating that Pin1 may promote hepatocarcinogenesis [9-12].

It has been shown that HDAC3 can be the substrate of Pin1 and 14-3-3 proteins [13]. 14-3-3, a family of conserved regulatory molecules in the regulation of mitogenic signal transduction, apoptotic cell death, and cell cycle, can protect HDAC3 form degradation, whereas Pin1 promotes HDAC3 degradation [13]. Therefore, the level of HDAC3 is regulated by competition between Pin1 and 14-3-3 proteins. Our previous study has shown that ZBP-89 could bind to HDAC3 protein to inhibit its deacetylation activity in HCC [2]. Thus it appears that HDAC3 is subjected to the control by both ZBP-89 and Pin1. Interestingly, the functions of both ZBP-89 and Pin1 are closely associated with NF-κB, as Pin1 can stabilize the NF-κB subunit p65 to enhance its activity and ZBP-89 can compete with NF-κB to bind to the promoter of certain genes such as MMP-3 [14-18]. We therefore hypothesize that NF-κB may play a role in the regulation of HDAC3 by ZBP-89 and Pin1 in HCC. The current study investigated the mechanism how ZBP-89 downregulated HDAC3 in HCC.

## Methods

### Cell lines, chemicals and reagents

The human HCC cell lines PLC/PRF/5 and HepG2 were obtained from American Type Culture Collection (Rockville, MD). Pin1-knockout cell line Pin1^−/−^ and wild-type Pin1^+/+^ cell line were a generous gift from Dr. Zi-Gang Dong (The Hormel Institute, University of Minnesota, MN) [19]. Pin1^−/−^ cells, derived from mouse embryo fibroblasts (MEFs), were originally generated from Pin1 knockout mice [20,21]. PLC/PRF/5, immortalized non-tumorigenic human hepatocyte cell line MIHA, Pin1^−/−^ and Pin1^+/+^ were maintained in Dulbecco’s modified Eagle medium (Invitrogen, Carlsbad, CA), and HepG2 was maintained in Minimum Essential Medium (Invitrogen, Carlsbad, CA). CAY10576, MG132, HDAC3 and HDAC4 antibodies were purchased from Cayman Chemical (Ann Arbor, MI). NF-κB inhibitory peptide SN50 was form Calbiochem (Darmstadt, Germany). Antibodies against ZBP-89, p65 and Pin1 were obtained from Santa Cruz Biotechnology (Santa Cruz, CA). Phospho-HDAC3 (Ser424) and phospho-IκBα (Ser32/36) antibodies was from Cell Signaling Technology (Beverly, MA). Ad-ZBP-89 viral vector was a generous gift from Dr. JL Merchant (University of Michigan, MI).

### Transfection of siRNA plasmid

The experiment was performed according to the previous publication [1]. Briefly, 3 × 10^5^ cells were seeded in 6-well plates 1 day prior to transfection. Pin1 siRNA sequence was constructed into mU6 siRNA vector which was a generous gift from Dr. Guoliang Huang (Guangdong Medical College, Guangdong, China). mU6-siPin1 or mU6-siControl plasmid was transfected into cells with Fugene reagent (Roche, Indianapolis, IN). Two days later, cells were lysed in RIPA buffer on ice with protease inhibitors.

### Cytosolic and nuclear extracts preparation

**Figure 1:**
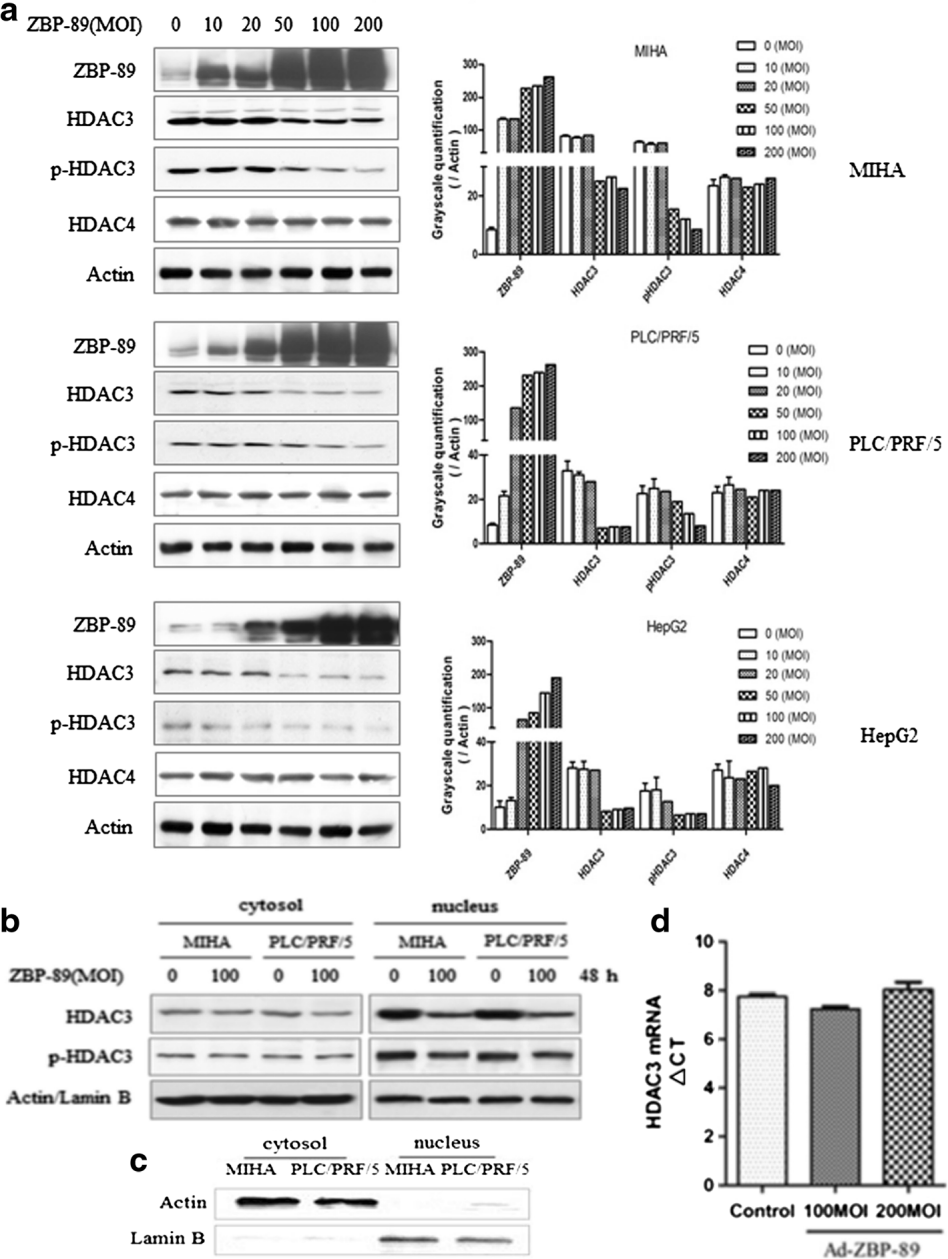
**Reduction of HDAC3 by ZBP-89.** The ectopic expression of ZBP-89 attenuated HDAC3 protein expression but not HDAC4 in MIHA, PLC/PRF/5 and HepG2 cells. The level of HDAC3 was decreased in a dose-dependent manner by ZBP-89 **(a)**. Cell lysate was obtained to separate the cytoplasmic and nuclear fractions. The reduction of HDAC3 and pHDAC3 proteins mainly occurred in the nucleus **(b)**. The cytoplasmic and nuclear fractions were verified using anti-actin and anti-lamin B antibodies respectively **(c)**. RT-PCR analysis showed that the level of HDAC3 mRNA was not changed by ZBP-89 ectopic expression **(d)**. All experiments replicated at least three times.

A nuclear extraction kit (Panomics, Fremont, CA) was used to isolate nuclear and cytoplasmic fractions and it was done according to our previous publication [22]. For details, please refer to Additional file 1. The isolated nuclear and cytoplasmic fractions were confirmed by Western blot using anti-actin and lamin B antibodies respectively (Figure 1c).

### Co-immunoprecipitation (Co-IP) and western blot

Co-IP was carried out according to the previous report with some modification [23]. Briefly, cell lysates were precleared with protein A/G Plus-agarose beads at 4°C for 4 h. Meanwhile, the primary antibody was coupled with Dynabeads Protein G beads (Invitrogen, Carlsbad, CA) at room temperature for 30 min. The precleard cell lysates were incubated with beads-coupled primary antibody overnight at 4°C. The antibody-antigen immunocomplex was collected though magnetic separation and washed with PBS for three times. Immunocomplexes were released by boiling in 2 × SDS sample buffer. 10% SDS-PAGE gels were used and proteins were transferred onto Hybond membranes (GE Healthcare, Arlington Heights, IL) by electroblotting at 100 V for 2 h. After blocking with 5% nonfat milk, the membrane was incubated with the designated primary antibody for 2 h, followed by incubation with a HRP-conjugated secondary antibody for 1 h. The signal was visualized by ECL reagent.

### Reverse transcription and real-time PCR

Total mRNA was extracted by a commercial mRNA extraction kit (Invitrogen, Carlsbad, CA ). 2 μg of RNA were reverse-transcripted into cDNA using oligo dT primers. Real-time PCR primers for HDAC3 were synthesized by Invitrogen. Forward primer: GCTGGAGGGAAAAGGAGTGG; Reverse primer: GGCCTTGGGAGAGAGAGGAA. PCR was carried out at 94°C for 30 s, 55°C for 30 s and 72°C for 45 s for 30 cycles, using ABI prism 7700 sequence detector and software to analyze the data (Applied Biosystems, CA).

### Immunohistochemistry and immunofluorescence

The tissue sections were blocked with SuperBlock buffer (Thermo, IL) for 30 min, then incubated with a primary antibody overnight at 4°C, followed by incubation with a HRP-labeled IgG secondary antibody with three washes in interval. The antigen-antibody complex was visualized by the diaminobenzidine method. PBS that replaced the primary antibody was used as a negative control. For immunofluorescent staining, cells were seeded on the Millicell EZ SLIDE (Millipore, MA) and incubated overnight. After treatments, the cells were fixed with 4% paraformaldehyde for 10 min, followed by washing in 0.1% Triton X-100 with shaking for 10 min and then blocking with 2% BSA for 30 min. The primary antibody was diluted with 2% BSA and incubated with cells at 4°C overnight, followed by incubating with FITC- or Rhodamine-labeled secondary antibody for 1 h. Finally, Prolong Gold antifade reagent (Invitrogen, Carlsbad, CA) with DAPI stained the cells for 5 min. Fluorescent cells were visualized using the Zeiss Axioplan 2 microscope (Zeiss, Germany).

### Xenograft animal model

The xenograft tumor model was generated by injecting 2 × 10^6^ PLC/PRF/5 cells into the left axilla nude mice for three weeks. Tumor-bearing mice were randomly divided into two groups, 10 mice per group. The mice in the therapeutic group were injected with ZBP-89 viral expression vector (1 × 10^10^ pfu) into tumor tissues, while the mice in the control group received saline injection. The first injection was done at the first day when tumor-bearing mice were divided into two groups, and the second injection was done at 7 days later. Two weeks after the 2^nd^ injection, the tumors were excised intact and weighted. Tumor tissues were subjected to H&E staining and immunohistochemical staining. The tissue lysate was analyzed by Western blot to detect ZBP-89, HDAC3 and Bak expression. The welfare and experiment protocol of these mice were strictly followed according to the guidelines approved by the Animal Experimentation Ethics Committee of our institute.

### Statistical analysis

Analyses were performed using the Statistics Package for Social Sciences (SPSS for Windows, version 13.0, SPSS). One-way ANOVA was used to compare means of three or more samples. *t*-test or paired *t* test was used to compare the means of two variables. P values of less than 0.05 were considered statistically significant.

## Results

### The ectopic expression of ZBP-89 diminished HDAC3 but not HDAC4 expression

We found that ZBP-89 inhibited the expression of HDAC3 and pHDAC3 proteins but not HDAC4 (Figure 1a), confirming our previous finding [2]. ZBP-89 also did not affect the expression of pHDAC4 protein (Additional file 1: Figure S1). In this study, we further examined if ZBP-89 could change subcellular distribution of HDAC3. After different dose- and time-exposures to ZBP-89, cytosol and nuclear extracts were respectively collected and subjected to Western blot. Results indicated that the decrease of HDAC3 and pHDAC3 proteins mainly occurred in the nucleus (Figure 1b). We further found that ZBP-89 did not change the level of HDAC3 mRNA as evident by RT-PCR analysis (Figure 1d), suggesting that ZBP-89 downregulates HDAC3 at the post-translational instead of transcriptional level.

### Knockdown of Pin1 greatly blocked ZBP-89-mediated HDAC3 reduction

**Figure 2:**
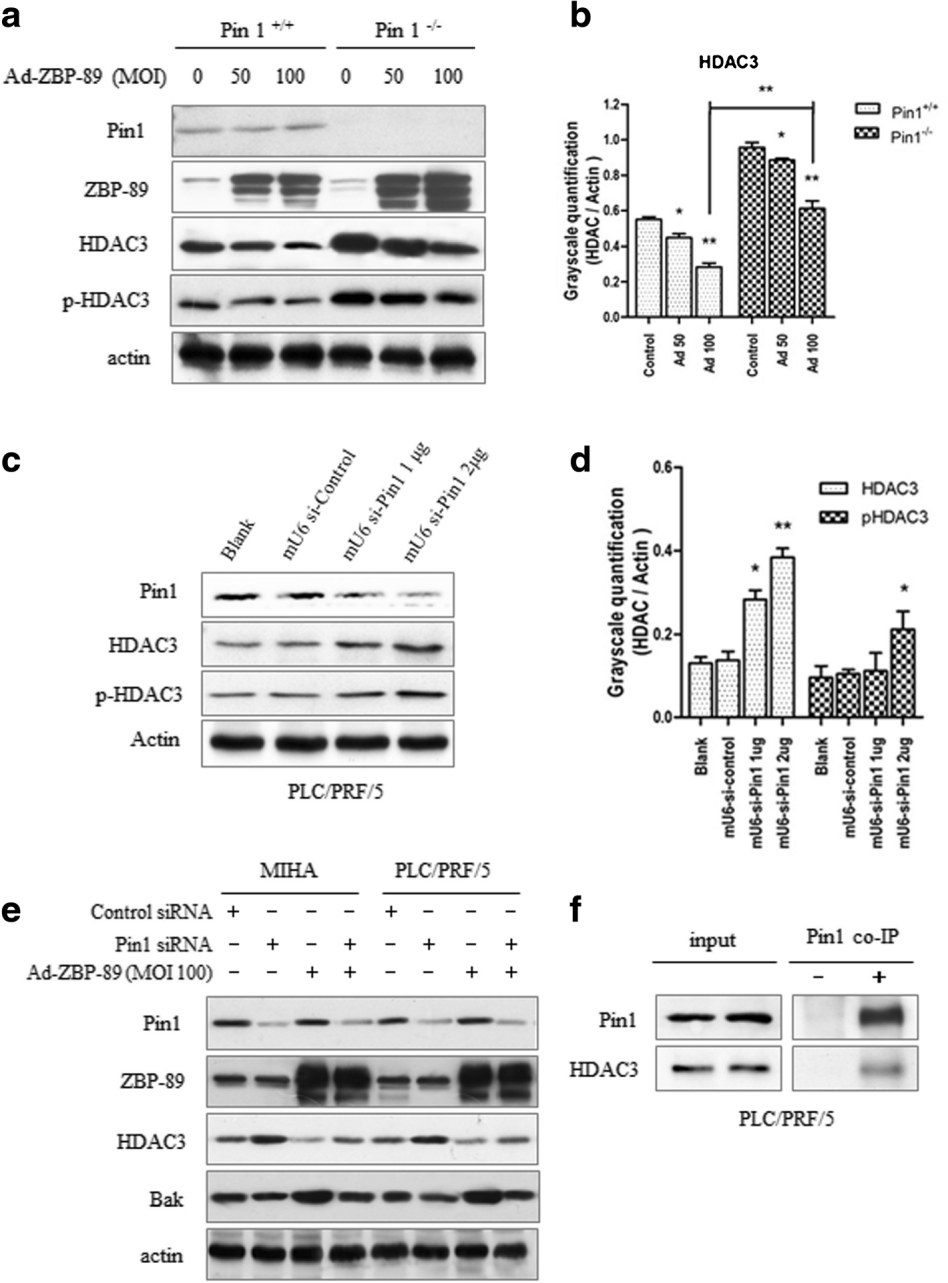
**Knockdown of Pin1 expression inhibited ZBP-89-mediated reduction of HDAC3.** Pin1 allele-knockout Pin1^−/−^ and Pin1^+/+^ cells were infected with Ad-ZBP89 and the levels of HDAC and pHDAC were examined by Western blot **(a)**, The densities of protein bands were determined and the ratio of test (HDAC) to the control (actin) was calculated **(b)**. This ratio was significantly lower in Pin1^+/+^ cells than in Pin1^−/−^ cells. Pin1^+/+^ represents Pin1^+/+^ MEFs, and Pin1^−/−^ represents Pin1^−/−^ MEFs. Cells were treated with Pin1 siRNA to inhibit Pin1 expression. The inhibition of Pin1 expression increased the levels of HDAC3 and pHDAC3 in PLC/PRF/5 cells **(c)**. The densities of protein bands were determined to quantitate the protein levels **(d)**. MIHA and PLC/PRF/5 cells were pretreated with Pin1 siRNA before Ad-ZBP-89 infection. Pin1 inhibition significantly inhibited ZBP-89-mediated reduction of HDAC3 **(e)**. The up-regulated expression of Bak by ZBP-89 was also markedly suppressed by Pin1 siRNA **(e)**. Co-IP experiments showed that Pin1 bound to HDAC3 in PLC/PRF/5 cells **(f)**. All experiments replicated at least three times. *p < 0.05, **p < 0.01 paired *t* test, compared with respective controls.

To study the role of Pin1 in the ZBP-89-mediated HDAC3 reduction, two approaches were used. Firstly, we examined the expression of HDAC3 in Pin1 allele-knockout JB6 C141 Pin1^−/−^ and Pin1 wild-type cell lines JB6 C141 Pin1^+/+^, and found that the reduction of HDAC3 by ZBP-89 was much more obvious in Pin1^+/+^ cells than in Pin1^−/−^ cells (Figures 2a and 2b), suggesting that ZBP-89-mediated HDAC3 reduction is partially but not totally dependent on the presence of Pin1. It also showed that Pin1^−/−^ cells had higher levels of HDAC3 and pHDAC3 than that in Pin1^+/+^ cells. Secondly, we used Pin1 siRNA to block the expression of Pin1 and then examined HDAC3 expression in MIHA and PLC/PFR/5 cells. Pin1 knockdown increased the levels of HDAC3 and pHDAC3 (Figures 2c and d). Further experiments showed that the block of Pin1 expression suppressed ZBP-89-mediated HDAC3 reduction in both cells (Figure 2e). Accompanied with the attenuation of ZBP-89-mediated HDAC3 reduction, ZBP-89-induced Bak was inhibited (Figure 2e). Finally, the co-IP experiment showed that Pin1 was able to bind to HDAC3 (Figure 2f), suggesting that Pin1 may directly interact with HDAC3.

### ZBP-89 reduced the level of IκB

**Figure 3:**
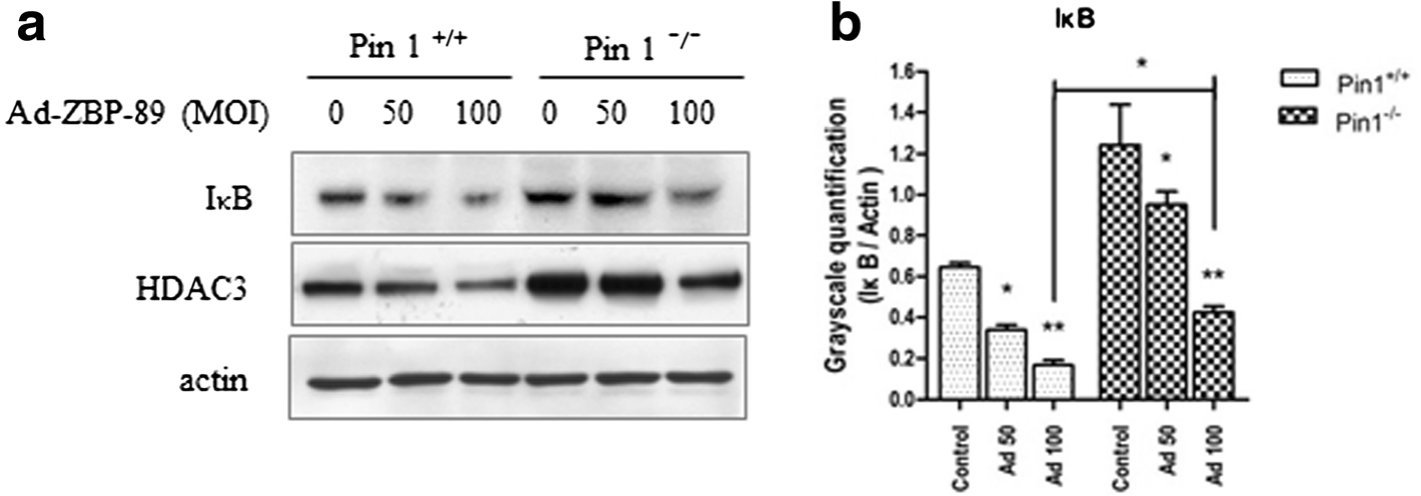
**ZBP-89 stimulated IκB phosphorylation to decrease HDAC3.** Cells were infected with Ad-ZBP-89. Total protein was isolated and subjected to Western blot analysis of IκB and HDAC3 **(a)**. ZBP-89 reduced the level of IκB in a dose-dependent manner in HCC cells. The densities of IκB protein band were determined and the ratio of test (IκB) to the control (actin) was calculated **(b)**. This ratio was significantly lower in Pin1^+/+^ cells than in Pin1^−/−^ cells (*p < 0.05, **p < 0.01, paired *t* test, compared with respective controls.

To examine the role of IκB, an endogenous inhibitor of NF-κB, in the ZBP-89-mediated HDAC3 reduction, Western blot was used to determine the level of IκB in cells treated with Ad-ZBP-89. It was found that the ectopic expression of ZBP-89 reduced IκB protein in both Pin1^+/+^ and Pin1^−/−^ cells (Figure 3a). However, the reduction was much greater in Pin1^+/+^ cells than in Pin1^−/−^ cells as the ratio of the reduction is significantly lower in the former than in the latter (Figure 3b). This finding indicates that ZBP-89-induced reduction of IκB is partially associated with the presence of Pin1.

### IκB degradation inhibitors suppressed ZBP-89-meditaed HDAC3 reduction

**Figure 4:**
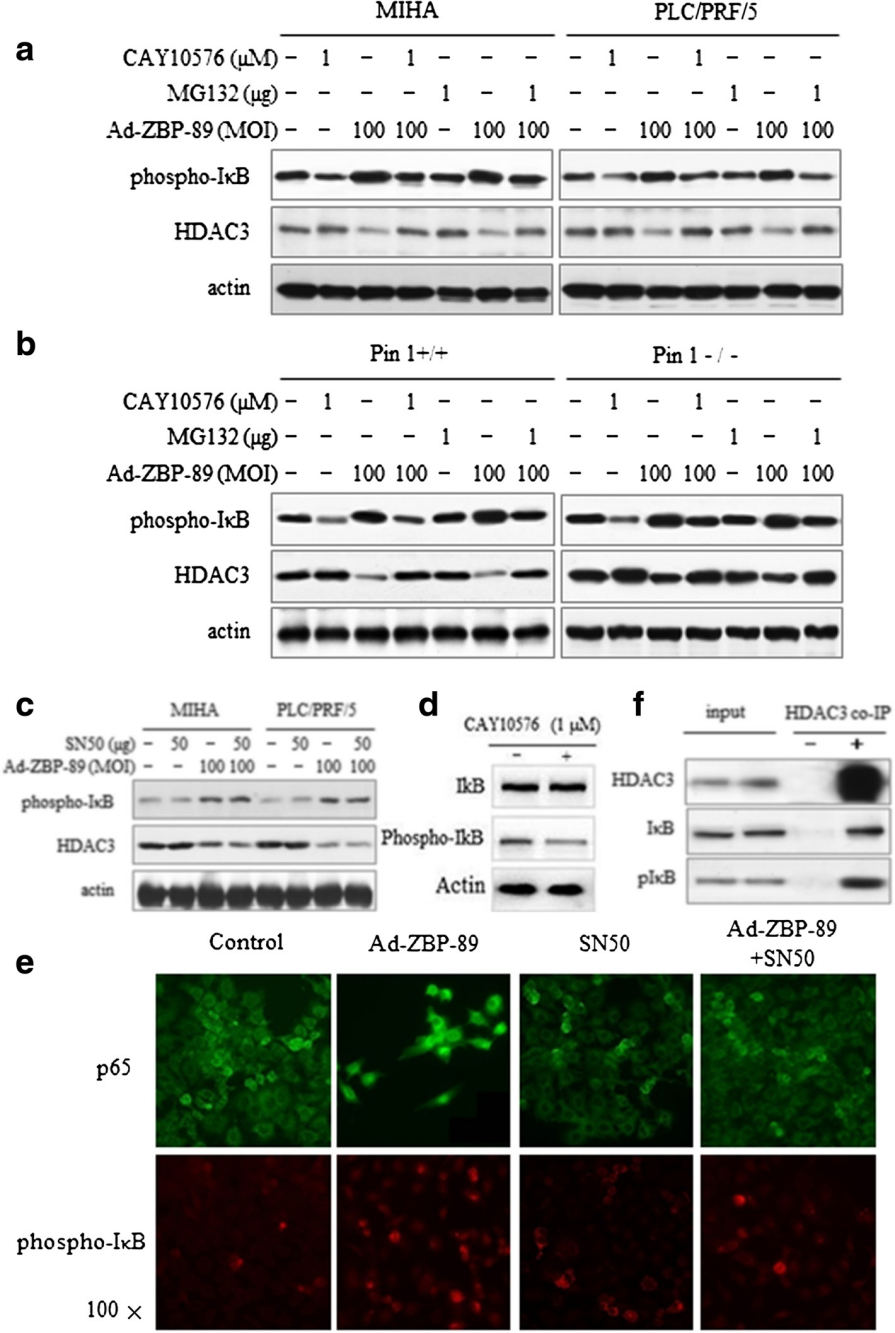
**ZBP-89 reduced HDAC3 via stimulating IκB phosphorylation.** After cells were infected with Ad-ZBP-89, they were treated with CAY10576, MG132 and SN50. After the treatment, total protein was isolated and subjected to Western blot analysis for pIκB and HDAC3. Either CAY10576 or MG132 inhibited pIκB as well as HDAC3 in MIHA, PLC/PRF/5 and Pin1^+/+^ and Pin1^−/−^ cells **(a** and **b)**. p65/p50 inhibitory peptide SN50 suppressed NF-κB activity **(e)** but had no effect on pIκB **(c)**. The level of ZBP-89 mediated HDAC3 reduction was also not affected by SN50 **(c)**. CAY10576 did not affect the level of total IκB **(d)**. Co-IP and Western blot were employed to test if IκB and pIκB could bind to HDAC3. IκB and pIκB were both detected in the immunoprecipitated HDAC3 complex, indicating that HDAC3 can form a complex with IκB, as well as pIκB **(f)**.

To explore how IκB was reduced by ZBP-89 and participated in the reduction of HDAC3, three different NF-κB inhibitors were employed to treat cells and the level of pIκB was determined. It is known that IκB phosphorylation can lead to the degradation of IκB [24]. CAY10576 is a selective inhibitor of IKK-ε whose activation can lead to the degradation of IκB and thus the activation of NF-κB [25]. MG132, a proteasome inhibitor, can block the activation of NF-κB through preventing proteasome-mediated degradation of IκB [24]. SN50 is a specific inhibitor of p50/p65 nuclear translocation and transcriptional activity but does not interfere with IκB degradation [26,27]. Results showed that pretreatment with CAY10576 inhibited ZBP-89-mediated induction of pIκB in MIHA, PLC/PRF/5, Pin1^+/+^ and Pin1^−/−^ cells (Figures 4a and b). Similar results were obtained when the proteasome inhibitor MG132 was used. SN50 inhibited NF-κB but did not affect the level of pIκB (Figures 4c and e). Nevertheless, similar to SN50, both CAY10576 and MG132 could inhibit ZBP-89-mediated p65/50 (data not shown). However, importantly the ZBP-89-mediated HDAC3 reduction could be prevented by CAY10576 or MG132 (Figures 4a and b), but not by SN50 (Figure 4c). Therefore, these data appear to suggest that the changes in IκB or pIκB rather than the activity of NF-κB are involved in ZBP-89-mediated HDAC3 reduction. Using co-IP and Western blot analysis, we testified that both IκB and pIκB were able to bind to HDAC3 (Figure 4f), suggesting a direct interaction between both proteins in HCC cells.

### ZBP-89 decreased HDAC3 but increased Bak in xenograft tumor tissues

**Figure 5:**
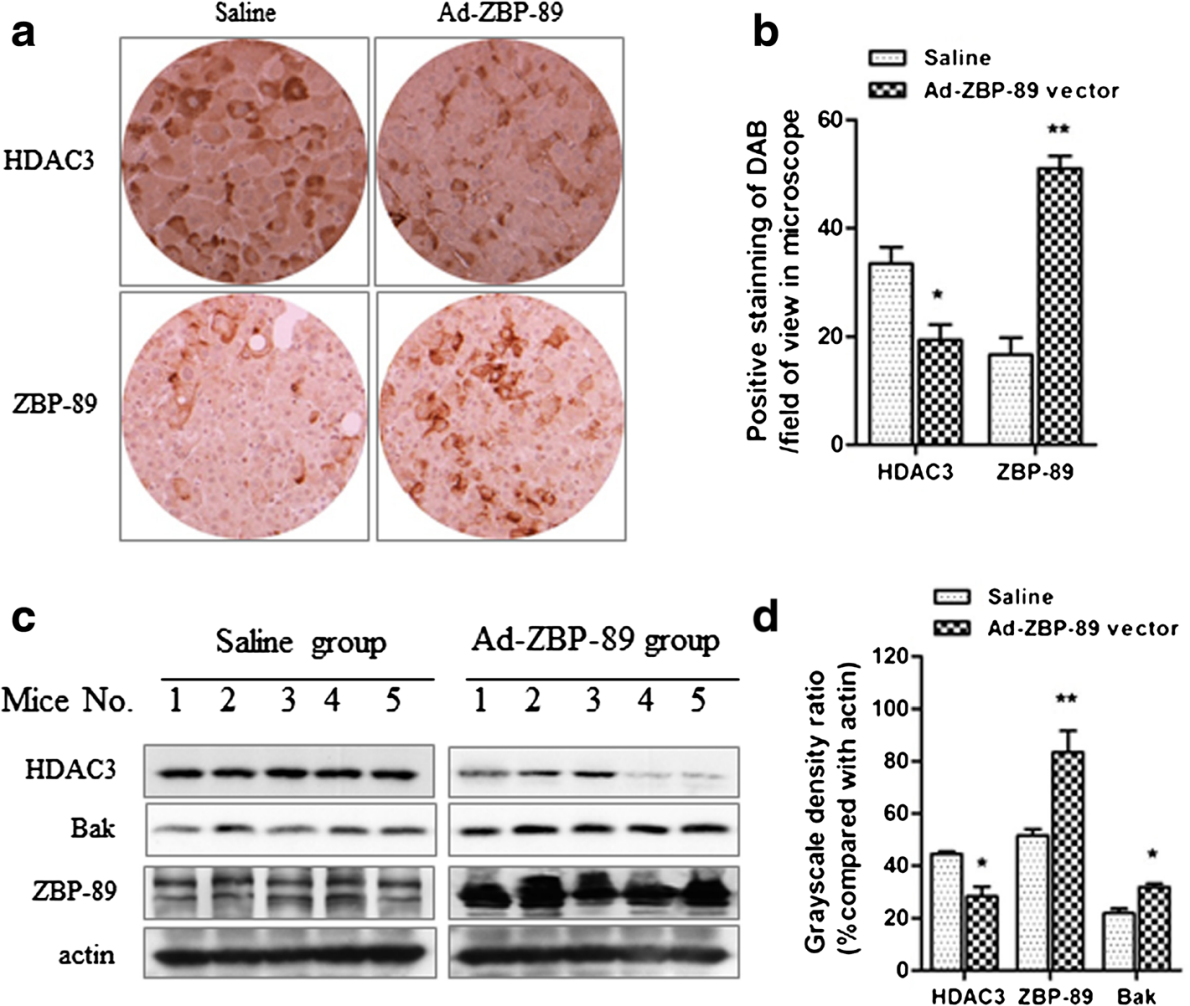
**ZBP-89 decreased HDAC3 but increased Bak in xenograft tumor tissue.** Ad-ZBP-89 infection effectively enhanced ZBP-89 levels in 10 tumor-bearing mice. The expression of HDAC3 in xenograft tumor tissues was examined by both immunohistochemical staining and Western blotting analysis **(a and c)**. The positive-stained cells were counted and the density of Western blot bands was determined **(b and d)**. Both methods showed that ZBP-89 overexpression significantly reduced HDAC3 expression, compared with saline controls (*p < 0.05, **b** and **d**). The ectopic expression of ZBP-89 also significantly increased Bak expression (*p < 0.05, **d**). **p < 0.01, compared with saline controls.

A nude mouse tumor model was established by subcutaneous injection of PLC/PRF/5 cells. The immunohistochemical staining of tumor tissues showed that the positive staining of HDAC3 in Ad-ZBP-89 group was less than that in the saline group. We randomly counted the positive-stained cells in 7 fields in each section, the statistical analysis revealed that the expression of HDAC3 was significantly decreased in mice treated with Ad-ZBP-89 compared with those treated with saline (Figures 5a and b). The immunohistochemical result was confirmed by Western blot analysis, as the level of HDAC3 protein was lower in mice treated with Ad-ZBP-89 than those treated with saline (Figures 5c and d), but HDAC4 level did not show obvious difference (data not shown). Our data also confirmed that ZBP-89 could significantly increase the level of Bak (Figure 5c and d).

## Discussion

In this study, we first confirmed our previous finding that ZBP-89 reduced both HDAC3 and pHDAC3 proteins in HCC cells [2]. The level of HDAC3 is frequently increased in HCC [5-7], suggesting a possible role of HDAC3 in hepatocarcinogenesis. Indeed, the increased expression of HDAC3 is significantly correlated with DNA copy number gains in HCC [5], and HDAC3 acts on miR-224-residing locus in Xq28 to increase the level of miR-224, one of the most commonly up-regulated microRNAs in HCC [6]. The positive role of HDAC3 in the growth and progression of HCC is further supported by the inhibitory experiment, in which the downregulation of HDAC3 results in a strong anti-tumor effect and enhances the efficacy of chemotherapeutic agents in HCC [5-7]. HDAC3 may also serve as a biomarker for HCC recurrence as the high expression level of HDAC3 is associated with the reduced recurrence-free survival of HCC patients [7]. Although HDAC3 participates in the development, progression, treatment and survival of HCC, the molecular mechanism leading to its increase in HCC is unknown. ZBP-89 is known as an apoptotic inducer in HCC and studies have demonstrated that ZBP-89 can enhance the pro-apoptotic Bak [1.2]. It is not surprising to find that ZBP-89 is able to reduce HDAC3 since there is evidence showing that the function of ZBP-89 is related to epigenetic events. For example, ZBP-89-induced p21waf1 activation can be enhanced by HDAC inhibitor sodium butyrate [28]. ZBP-89 and HDAC3 can form a complex to help HDAC3 bind to the p16 promoter, resulting in p16 downregulation [29].

It appears that there are some connections between HDAC3 and NF-κB or its subunits. NF-κB subunit p65 has been shown to recruit HDAC3 to the antioxidant response element of Nrf2 gene, leading to local histone hypoacetylation [30]. The activity of NF-κB is associated with the nuclear export of HDAC3, and HDAC3 can efficiently inhibit CREB3-induced NF-κB activation [31,32]. The activation of NF-κB by IκB phosphorylation is accompanied with the reduction in HDAC levels/activity [33]. IκBα is known to bind HDAC3 through its ANKYRIN amino acid repeats [34]. We therefore wonder whether NF-κB or its subunits contribute to ZBP-89-induced the reduction of HDAC3 in HCC. To address this question, we first demonstrated that accompanied with the reduction of HDAC3, ZBP-89 could enhance IκB phosphorylation to reduce the level of IκB in HCC. We further showed that ZBP-89 could form a complex with either IκB or pIκB, suggesting a possible direct interaction between them. These findings appear to imply that ZBP-89 promotes IκB degradation via enhancing IκB phosphorylation (pIκB) to activate NF-κB, which may lead to the reduction of HDAC3. To verify this assumption, we employed three different inhibitors to block NF-κB activity. CAY10576 and MG132 can prevent the degradation of IκB to block the activation of NF-κB [24,25]. SN50 inhibits p50/p65 nuclear translocation rather than IκB degradation to block the activation of NF-κB [26,27]. Unexpectedly, though all these three inhibitors can block the activation of NF-κB, ZBP-89-mediated HDAC3 reduction can be prevented by CAY10576 or MG132 only, but not by SN50. Therefore, the decrease of IκB or the increase pIκB rather than the elevated activity of NF-κB contributes to ZBP-89-mediated HDAC3 reduction in HCC.

To further explore the relevant mechanism of ZBP-89-mediated the reduction of HDAC3, we studied the role of Pin1 in this pathway since Pin1 can bind to HDAC3 to cause HDAC3 degradation [13], and Pin1 is overexpressed in HCC [9-12]. We found that the inhibition of Pin1 increased the level of IκB and that Pin1-knocked-out cells Pin1^−/−^ possessed higher levels of HDAC3 and pHDAC3 than cells with Pin1 wild-type cells Pin1^+/+^, indicating that Pin1 may negatively regulate HDAC3 via decreasing the level of IκB. We further demonstrated that the downregulation of Pin1 significantly, though not completely, inhibited ZBP-89-mediated HDAC3 decrease. The similar results were also found in Pin1^−/−^ and Pin1^+/+^ cells. The inhibitory effect of ZBP-89 on HDAC3 was much stronger in the Pin1^+/+^ cells than in Pin1^−/−^ cells. These findings suggest that ZBP-89-mediated HDAC3 reduction is partially dependent on the Pin1 protein whose level is negatively associated with IκB. Therefore, Pin1 is required for the maximal effect of ZBP-89 on HDAC3 reduction.

Our study suggests that ZBP-89-mediated HDAC3 reduction likely contributes to its ability to increase pro-apoptotic molecule Bak in HCC cells. First, when cells were treated with ZBP-89, the level of HDAC3 protein was negatively associated with the expression of Bak protein in cell culture experiments. Second, the inhibition of Pin by siRNA resulted in the increase of HDAC3 but the decrease of Bak in the presence of ZBP-89. Third, in the mouse model of HCC, ZBP-89 treatment led to the reduction of HDAC3 but the increase of Bak. The close association between HDAC3 and Bak in ZBP-89-treated cells is in line with the finding that the inhibition of HDAC3 leads to the increase of Bak [35].

## Conclusions

The study demonstrates that ZBP-89-mediated reduction of HDAC3 is partially dependent on the phosphorylation of IκB and the presence of Pin1, but is independent of NF-κB activity. The reduction of HDAC3 likely contributes to ZBP-89-induced increase of Bak. The novel pathway identified herein may open up a new field to inhibit HCC by the application of ZBP-89 and the inhibition of HDAC3.

## Additional file

Additional file 1:
**Supplemental data.**

## Abbreviations

Co-IP: Co-immunoprecipitation
HCC: Hepatocelluar carcinoma
HDAC3: Histone deacetylase 3
IκB: I kappa B
Pin1: Peptidyl-prolyl cis/trans isomerase 1
PBS: Phosphate-buffered saline
