# Supplementary material for: ZBP-89 reduces histone deacetylase 3 by degrading IkappaB in the presence of Pin1
Source: J Transl Med. 2015 Jan 27;13:23. doi: 10.1186/s12967-015-0382-7 (PMC4311446; doi:10.1186/s12967-015-0382-7)

**Supplemental data**

**Methods**

**Cytosolic and nuclear extracts preparation**

5x10^6 cells were collected from cell cultures using a cell scraper or by trypsinization. Cell pellets were obtained by centrifugation at 250 x g for 5 min to discard supernatant. The packed cell volume was estimated, and 10 volumes of Cell lysis buffer (with protease inhibitors and DTT) were added to 1 volume of packed cells. The cells were vortexed for 10 s, set on ice for 10 min and vortexed again. The cytoplasmic fraction was separated from nuclei by centrifugation at 500 x g for 7 minutes at 4°C. The supernatant (cytoplasmic protein extract) was carefully removed to a new tube. The clear cytoplasmic protein was extracted by centrifuging at 20,000 x g for 15 min at 4°C, and the supernatant was transferred to a new tube. The cytoplasmic protein can be used directly or stored at -70°C for later analysis. 500 μl of the Nuclei washing buffer (with protease inhibitors and DTT) was added to the nuclear pellet, vortexed briefly and set on ice for 2 min. The supernatant was carefully removed by centrifuging at 500 x g for 7 minutes at 4°C. 150 μl of ice cold Nuclei storage buffer (with protease inhibitors and DTT) was added to the nuclei pellet. Pipette up and down 5-10 times to break up any clumps. Proceed with lysis or store frozen at -70°C. Add 1/10 volume of the Nuclei lysis reagent to the nuclear suspension. The tube was vortexed briefly and shaken for 15 min at 4°C (900-1200 rpm). Clear nuclear lysate was obtained by centrifugation at 20,000 x g for 5 min at 4°C. The supernatant (nuclear protein extract) was transferred to a new tube. The nuclear protein can be used directly or stored at -70°C for later analysis. The isolated nuclear and cytoplasmic fractions were confirmed by Western blot using anti-actin and lamin B antibodies respectively (Fig 1c).

**Supplemental Fig S1**. **No effect of ZBP-89 on the expression of pHDAC4.** PLC/PRF/5 cells were transfected with different doses of Ad-ZBP-89. Cell lysates were obtained and subjected to Western blot to detect pHDAC4.


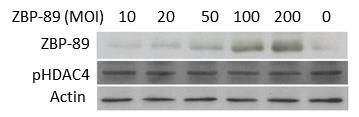

Supplement: Additional file 1: — Supplemental data. [file 12967_2015_382_MOESM1_ESM.docx]
